# Frameworks for self-management support for chronic disease: a cross-country comparative document analysis

**DOI:** 10.1186/s12913-018-3387-0

**Published:** 2018-07-25

**Authors:** Selena O’Connell, Vera J. C. Mc Carthy, Eileen Savage

**Affiliations:** School of Nursing & Midwifery, Brookfield Health Sciences Complex, University College Cork, Cork, Ireland

**Keywords:** Document analysis, Health policy analysis, Self-management support, Chronic disease

## Abstract

**Background:**

In a number of countries, frameworks have been developed to improve self-management support (SMS) in order to reduce the impact of chronic disease. The frameworks potentially provide direction for system-wide change in the provision of SMS by healthcare systems. Although policy formulation sets a foundation for health service reform, little is currently known about the processes which underpin SMS framework development as well as the respective implementation and evaluation plans.

**Methods:**

The aim of this study was to conduct a cross-country comparative document analysis of frameworks on SMS for chronic diseases in member countries of the Organisation for Economic Cooperation and Development. SMS frameworks were sourced through a systematic grey literature search and compared through document analysis using the Health Policy Triangle framework focusing on policy context, contents, actors involved and processes of development, implementation and evaluation.

**Results:**

Eight framework documents published from 2008 to 2017 were included for analysis from: Scotland, Wales, Ireland, Manitoba, Queensland, Western Australia, Tasmania and the Northern Territory. The number of chronic diseases identified for SMS varied across the frameworks. A notable gap was a lack of focus on multimorbidity. Common courses of action across countries included the provision of self-management programmes for individuals with chronic disease and education to health professionals, though different approaches were proposed. The ‘actors’ involved in policy formulation were inconsistent across countries and it was only clear from two frameworks that individuals with chronic disease were directly involved. Half of the frameworks had SMS implementation plans with timelines. Although all frameworks referred to the need for evaluation of SMS implementation, few provided a detailed plan.

**Conclusions:**

Differences across frameworks may have implications for their success including: the extent to which people with chronic disease are involved in policy making; the courses of action taken to enhance SMS; and planned implementation processes including governance and infrastructure. Further research is needed to examine how differences in frameworks have affected implementation and to identify the critical success factors in SMS policy implementation.

**Electronic supplementary material:**

The online version of this article (10.1186/s12913-018-3387-0) contains supplementary material, which is available to authorized users.

## Background

Self-management is a process through which individuals actively cope with their chronic disease in the context of their daily lives [1]. Supporting individuals to self-manage is an important strategy to reduce the burden of chronic disease [2, 3]. Self-management support (SMS) has had positive effects on health outcomes for people with chronic disease including increased health related quality of life [4–6]. In some countries, governments have developed health policy to support self-management of chronic disease in order to promote positive health outcomes.

SMS policy and frameworks, such as those developed in Australia [7] and Ireland [8], aim to guide system-wide changes in service delivery for chronic disease management. However, healthcare policies have not always achieved their aims and implementation targets [9, 10]. Health policy is a complex phenomenon [11]. Although there is little guidance on how to conduct analyses of health policies, the use of a framework has been recommended [11]. A commonly used framework is the Health Policy Triangle which comprises of four components: context, content, actors, and processes [12–14]. The *content* of a policy refers to the substance of the policy while the *context* relates to the systemic factors which can affect policy such as political, economic, social, national and international influences [13]. The *actors* are the stakeholders who influence policy, including individuals, organisations and government while the *process* concerns the ways in which policies are initiated, developed, negotiated, implemented and evaluated [13].

There is potential for considerable heterogeneity across chronic disease SMS policies from different countries. For example, there are contextual factors that differ across health systems including funding; organisation and governance; and the service reforms for population health priorities such as chronic disease management [15–17]. The content of chronic disease management policy, such as priorities on courses of action, can vary with some countries emphasising the role of nursing in chronic disease SMS and others emphasising accessibility to specialist multidisciplinary teams in primary care [16]. Many approaches to providing SMS have been studied such as peer support and online programmes [5, 18, 19]. The evidence is not clear on which SMS approaches provide the greatest benefit to individuals with chronic disease [5, 20] which could potentially result in countries adopting different courses of action to support self-management.

In terms of actors, the engagement of a diverse range of stakeholders in health policy making is expected [21]. Patient and public involvement (PPI) is called for in the development of health strategies [22]. The potential benefits of PPI include services adapted to local needs, the possibility of identifying innovative approaches to the problem as well as giving a voice to marginalised groups [22]. Specific to SMS, while research has examined patients’ experiences of self-managing their chronic disease, few studies have directly sought patients views regarding desired outcomes [23]. To date, little is known about patient involvement in SMS policy development.

The process of implementing SMS may be challenging. For example, there may be varying levels of readiness to endorse SMS as an approach to chronic disease management at the levels of the individual with chronic disease, the healthcare provider and the organisation [24]. A clear and strategic implementation plan is recommended for health policies and strategies [21, 25]. The World Health Organisation (WHO) outlined the importance of including governance arrangements, consideration for current capacity and resource planning, and mechanisms for evaluation in national policy and strategy documents [21]. Therefore, the level of detail in SMS frameworks implementation plans may affect the extent to which they achieve their intended outcomes.

Over the past decade, SMS frameworks for chronic disease have been developed as a policy initiative in various countries [7, 8, 26]. To the best of our knowledge, SMS frameworks have not been previously compared across countries. Therefore, the aim of this study was to conduct a cross-country comparative document analysis of frameworks on SMS for chronic diseases. It was aimed to compare countries in terms of policy content, the contexts influencing policy development, stakeholder involvement, and processes of policy development, as well as implementation and evaluation plans. Countries in the early stages of policy development and implementation can draw on this analysis.

## Methods

This study was designed as a comparative document analysis and adopted principles and procedures of systematic review methods. Document analysis, as a qualitative method, is “a systematic procedure for reviewing and evaluating documents” [27]. This procedure organises document information into categories which relate to specified research questions/categories [27]. Data collection and analysis were guided by the Health Policy Triangle [12]. SMS frameworks can be considered health policy documents in line with the definition of health policy provided by Buse and colleagues, that is, ‘courses of action (and inaction) that affect the set of institutions, organizations, services and funding arrangements of the health system’ [13]. As presented in Table 1, categories of information for analysis were constructed around the four areas of the Health Policy Triangle: context, content, processes and actors [12].

**Table 1:** Categories of information for analysis

Category	Descriptor	Examples/Application
Context	Factors which can influence policy development including past provision of SMS for chronic disease	Healthcare structures and governance, health service reform agenda, burden of chronic disease, demographic and prevalence trends of chronic disease, e-health trends, information on previous delivery of SMS
Content	Scope of the framework, defining features of SMS espoused, goal statements, policy statements of action	Chronic diseases targeted and scope of population for intervention; components of SMS definition; stated goal including intended effect and outcomes; SMS priorities or courses of action
Actors	Stakeholders involved in policy making	Government representatives, clinicians, health service managers, people with chronic disease, voluntary sector representatives and community and advocacy groups.
Processes	How information and actors interests are incorporated in policy formation; proposed process of implementation and evaluation	Evidence used and processes of stakeholder involvement such as consultation, involvement in workshops and surveys, member of working group; implementation plans, associated timelines, facilitation processes; evaluation strategies and feedback process.

### Inclusion criteria

Eligibility criteria included documents that: exclusively targeted SMS for chronic diseases in adult populations; targeted more than one chronic disease/condition; originated in countries which are members of the Organisation for Economic Co-operation and Development (OECD); were produced by/affiliated with a national government or national healthcare service (for countries such as USA, Australia, Canada and the United Kingdom, documents at province/state/territory level were searched); offered recommendations for the provision of SMS within the respective healthcare system; were published in English and in the period from October 31st 2007 to November 30th 2017. Only the most recent SMS framework document from countries was included.

### Search process

A search of grey literature sources was conducted to retrieve relevant documents. While no gold standard exists for grey literature searching, this study drew on the strategy used by Godin and colleagues [28] with the aim of ensuring that the search methods used were explicit, reproducible and identified all relevant documents. The search involved the following three groups of terms: (1) self-manage, self-management, self-managing; (2) long-term condition, chronic condition, chronic illness, chronic disease, non-communicable disease; (3) policy, framework, guideline, model, strategy and standard.

The search was carried out in early December 2017 and consisted of four parts. Firstly, it included grey literature databases which contained policy and government publications: OAIster, BIREME Virtual Health Library, World Health Organisation (WHO) Information Repository for Information Sharing, OECD iLibrary, Open Grey, Grey Literature Report, Canadian Electronic Library and Analysis and Policy Observatory. As the search specifications differed between sites, different search terms were used. See Additional file 1 for the specific search combinations used. Secondly, an advanced google search was completed using the search term combinations (Additional file 1). The first 10 pages of each search including different combinations of search terms were screened akin to previous work [28].

A third search was carried out on Ministry of Health/ Department of Health websites within OECD countries. Sites were searched using their relevant search box and manually where this did not exist. The search terms used were ‘self-manage’, ‘self-managing’ and ‘self-management’. Finally, documents were searched for by hand-searching the reference lists in the relevant retrieved documents.

### Study selection

Initial screening of the title and abstract/executive summary/overview was carried out by SOC. The titles and respective URLs of potentially relevant documents were entered into Excel and retained for full-text screening. Full texts of documents were assessed for inclusion by two authors independently (SOC, ES). Disagreements about eligibility were resolved by consensus and where necessary by a third reviewer (VMcC). The reason for exclusion was recorded.

### Data extraction and analysis

Relevant information from documents was charted in a data extraction table which was initially piloted with two documents. The information was extracted by one author (SOC). This was then independently cross-checked by ES and VMcC. The data extracted included geographical location, author and year of publication as well as information categories guided by the Health Policy Triangle Framework [12]. This framework also guided the analysis with findings presented according to the components of the Health Policy Triangle: context, content, actors, and process.

## Results

**Fig. 1:**
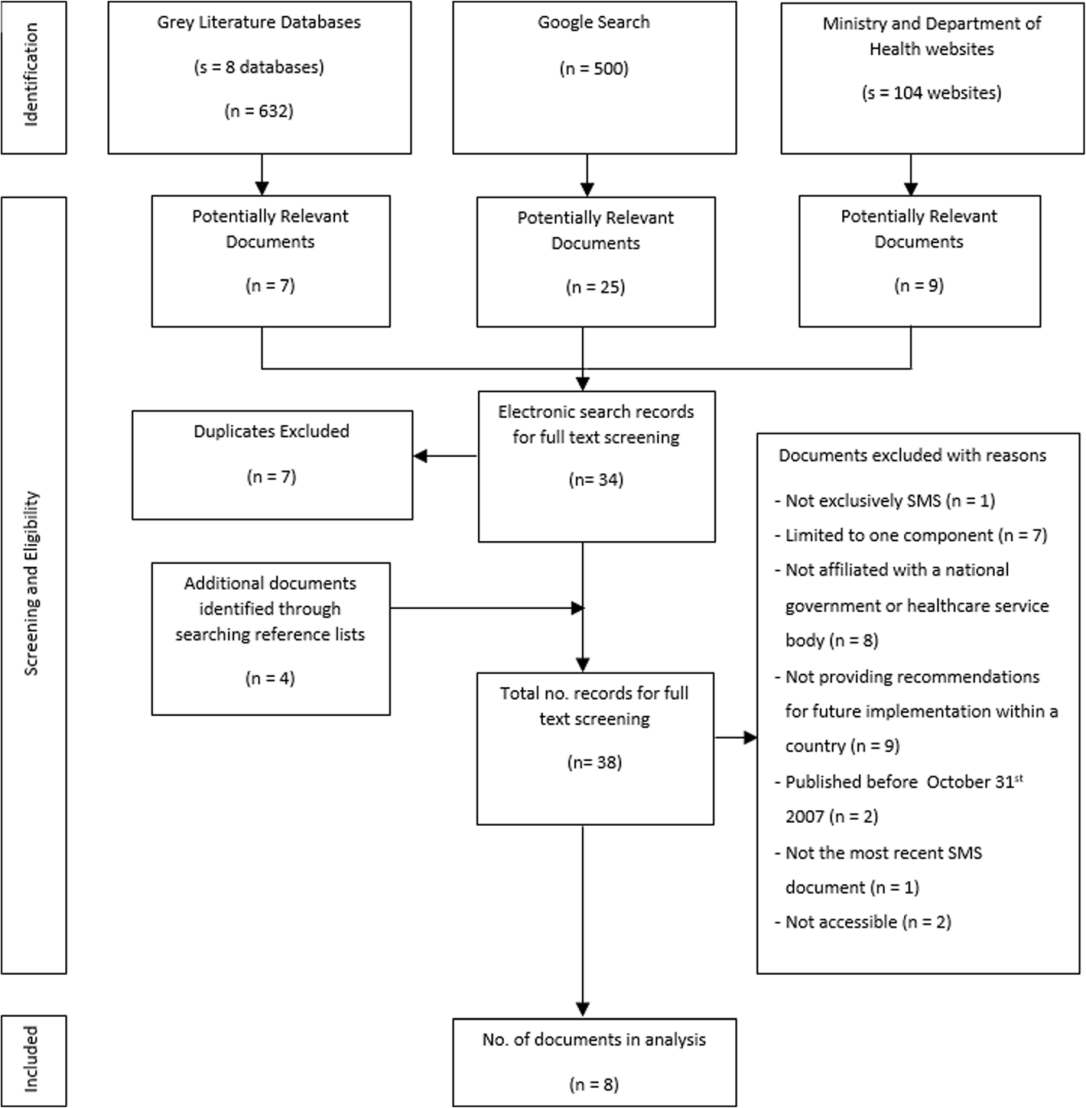
Document identification, screening and selection

Eight documents on SMS met the inclusion criteria (see flowchart in Fig. 1). The documents originated in Scotland (SL [29]), Wales (WL [30]), Ireland (IRL [8]), Manitoba, Canada (MB [31]) and four Australian states/territories: Queensland (QLD [7]), Western Australia (WA [26]), Tasmania (TAS [32]) and the Northern Territory (NT [33]). The frameworks were developed and published between 2008 and 2017. The full data extraction table is available in Additional file 2.

### Context

All eight frameworks highlighted the burden of chronic disease as context, referring to the impact of chronic disease in terms of: increased mortality; economic cost; use of health service resources, overburdened acute healthcare sectors; reduced participation in workforce; and impact on a person’s wellbeing. Apart from MB, all frameworks identified that self-management had been made a priority of previous strategies/policies pertaining to chronic disease management generally and/or the broader policy context of healthcare improvement and reform.

There was little data on the national healthcare systems and the influence of existing structures and governance on frameworks. SMS which was provided in the countries prior to developing a national framework was noted to be inadequate due to: variability across geographical areas (WL, IRL), with particular limitations in remote areas and for certain groups (MB, NT); involvement of different organisations and not well coordinated at whole system level (WL, NT); professionals not aware of services (WL, MB); and low rates of SMS from HCPs such as receiving written information on how to manage condition (WL, MB, IRL). For SL, it was noted that services did not place needs of individuals with chronic disease at the centre of care.

### Content

#### Scope of chronic disease supported

The extent to which the continuum of health was addressed by frameworks varied. IRL and SL focused mainly on self-management for individuals with a diagnosed chronic condition. QLD and NT addressed chronic diseases as well as risk factors while others covered the ‘continuum of care’ (WL, WA, MB, TAS). Reference to the ‘continuum of care’ differed across frameworks. It seemed to include risk identification through to disease management in MB compared to involving more active health promotion and disease prevention in WL and TAS.

As shown in Table 2, the range of chronic diseases differed across frameworks, although not explicit in three frameworks (SL, WL, MB). The IRL framework focused on a narrow range of conditions compared to other frameworks such as TAS which was found to target the widest range of conditions. SL, WL and IRL gave attention to multimorbidity or having more than one chronic disease.

**Table 2:** Chronic conditions addressed by frameworks

Framework	CVD	Diabetes	Respiratory	Renal	Musculoskeletal	Mental health	Cancers	Additional Information
QLD	X	X	X	X		X		
SL	–	–	–	–	–	–	–	Multimorbidity addressed.
WL	–	–	–	–	–	–	–	Multimorbidity addressed.
WA	X	X	X	X	X			Applicable to other conditions.
MB	–	–	–	–	–	–	–	
TAS	X	X	X		X	X	X	Additional conditions listed.
NT	X	X	X	X		X	X	Applicable to other conditions.
IRL	X	X	X					Multimorbidity addressed.

Note: conditions of focus were not explicit in SL, WL and MB

#### SMS defining features

All frameworks offered defining features of SMS, though the emphasis of the definitions varied somewhat. Two frameworks focused on the components of SMS: resources, programmes, services, tools (MB); education and interventions systematically provided (IRL). The remaining six frameworks referred to SMS in terms of where responsibility lies for providing it including healthcare systems, HCPs and social care providers, the community, and carers. Although the relationship between patients and HCP was noted as integral to SMS in all frameworks, the collaborative and shared responsibility was most explicit in some definitions (SL, WL).

#### Goal of framework

An overarching goal was explicit in five of the frameworks. These were: empowering and preparing individuals to actively manage their own healthcare (QLD, WA, NT), better outcomes for people with chronic diseases (TAS), participation of clients in healthcare ‘within their communities’ (WA); access to support (SL) and improvement of health services to support self-management (TAS, NT). Though not explicit, WL referred to coordination of care.

#### SMS priorities/courses of action

All eight frameworks included a range of strategies for SMS at individual, HCP, organisation and system levels. Some approaches to SMS were frequently prioritised. These included ensuring the provision of self-management skills and education programmes for people with chronic disease and providing education and training for HCPs (Table 3).

**Table 3:** Commonly prioritised courses of action to support self-management across frameworks

	Patient Education Programmes	Training for HCPs	Awareness raising	Accessibility of SMS	Technology to support SMS
QLD	Provide evidence based programmes	Provide education and training	Social marketing campaign	Provide suitable SMS	Consumer personal health record
WL	Generic and disease specific	Skills training	Provider awareness of programmes	SM information in various formats, signposting	Technology for reminders, self-monitoring, follow-up
WA	Coordinated SMS programmes and services	Curricula, professional development, mentoring	Marketing strategy, framework endorsement	Easy referral pathways, flexible delivery of services	Website for all stakeholders
MB	Telecare programme prioritised	–	Provider awareness of programmes	Research suitability for different groups	Online health portal
TAS	Make programs available	A range of training options, evidence-based practice	Provider awareness of programmes	Provide range of flexible resources	Range of resources including online
NT	Build capacity	Training and access to evidence-based practice	Framework endorsement	Clear referral pathways, SM information through various mediums	Electronic client record, SMS through online and other media
IRL	Map and increase provision of generic and disease specific	Curricula, professional development	SMS communication plan	Resources to account for health literacy, signposting	IT systems to support delivery, electronic patient record

Note: SL not included as it did not explicitly prioritise actions for implementation

The approaches to these common priorities varied and some frameworks had more clear and concrete courses of action than others. In all frameworks prioritising SMS programmes to educate and develop self-management skills of people with chronic diseases, there was an emphasis on increasing the availability of these services. Some frameworks were more specific in the content of the their programmes such as MB and IRL. The extent to which specific plans around training were detailed also differed across documents such as integration into undergraduate training curricula or continuing professional development.

Various courses of action were directed toward increasing the accessibility and appropriateness of SMS for different groups and individuals. Appropriate SMS for different groups was stated broadly as an action (QLD), or targeted through specific strategies. Some frameworks sought to increase healthcare provider awareness of patient programmes (WL, MB, TAS). Others proposed broader stakeholder and public awareness raising (QLD, WA, NT, IRL). In addition, frameworks emphasised different technological resources which could be used by stakeholders to support self-management (Table 3).

### Actors in framework development

There were differences across frameworks in the extent to which actors were detailed, the extent to which actors were involved as well as variability in the composition of actors (Table 4). The involvement of actors was not reported in WL or MB. The remaining six frameworks included actors and some form of working group. The working group actors were unclear in QLD. Otherwise the scope of the working group was usually small involving health division managers and/or HCPs. WA was the exception to this, involving all of its listed stakeholders in the working group.

**Table 4:** Policy actors and processes for each framework^a^

Framework	Actors	Development processes	Implementation Plans	Evaluation Plans
QLD	Members of strategy team and alliance, government, non-governmental organisations, professional bodies, private sector, consumer advocacy groups, HCPs universities and national and international ‘experts’	Consultation through forums, electronic questionnaire, feedback on drafts, meetings with key stakeholders. Evidence and existing frameworks.	Actions with timelines not reported. Proposed to be dynamic and flexible.	Plan/outcomes not detailed. Contains recommendations.
SL	Representatives from health and chronic disease organisations.	Working group and consultation on draft strategy.	Actions with timelines not reported. Processes of funding outlined.	Plan/outcomes not detailed. Contains recommendations.
WL	Not reported.	Not reported.	Actions with timelines not reported. Information on governance, infrastructure, incentives.	Plan/outcomes not detailed. Contains recommendations.
WA	Chronic disease consumers, carers, managers, policy developers, service providers, NGOs, researchers, self-management educators.	Consultation through an electronic qualitative survey, workshops with Strategy Review Group and feedback on draft.	Actions reported with timelines. Discussion of infrastructure, governance and funding.	Plan with timelines and actors responsible for leading evaluation.
MB	Not reported.	Guided by evidence, international and local.	Actions with timelines reported. Leading organisation and mechanisms of implementation identified.	Plan/outcomes not detailed. Contains recommendations
TAS	Reference group (HCPs and programme managers) and Chronic Conditions Clinical Network.	Consultation to identify priority areas. Use of specifically developed background paper and frameworks from other areas.	Actions with timelines not reported. Dynamic process, encourage uptake of resources by services and providers.	Plan/outcomes not detailed. Contains recommendations
NT	HCPs providing SMS, government, non-government and Aboriginal community controlled health service providers, HCPs and consumer groups.	Developed by working group. Used focus groups (urban and remote). Consultation on draft, evidence of experience and frameworks from other areas.	Actions reported with timelines.	Plan reported with performance measures for actions and timelines.
IRL	Health division and programme managers/ coordinators and researcher, HCPs, patient and chronic disease-specific organisations, people with chronic diseases, government, health service and university representation.	Working group and consultation on draft through focus groups, interviews. Evidence used from literature, international policy documents, national survey and forum for patient consultation.	Actions reported with timelines and actors responsible. Plans for governance, infrastructure and resourcing.	Early process measures detailed. Contains further recommendations.

^a^See Additional file 2 for further detail

It was explicit that people with chronic disease were involved in WA and IRL whereas in other countries, it was unclear if the involvement of consumer groups actually included people with chronic disease; although this was implied in the frameworks. In SL, it was emphasised that the framework was guided by people with long term conditions, though how this was achieved was not explicit.

### Process

#### Development

In six frameworks, a group or team was responsible for the development phase, as seen in Table 4 (QLD, SL, WA, TAS, NT, IRL). The process of framework development explicitly stated the use of consultation in all countries. Consultation involved forums (QLD, IRL), an electronic questionnaire (QLD, WA), meetings with stakeholders/focus groups (QLD, WA, NT, IRL), and feedback and refining of drafts. Explicit reference to the use of previous literature and evidence was articulated in QLD, MB, TAS, NT and IRL and most others appeared to reference previous literature, though SL appeared more limited in this area. Four frameworks mentioned drawing on frameworks already developed in other states/countries (QLD, MB, TAS, NT).

#### Implementation

Four frameworks included an implementation plan with specific actions associated with specific timeframes (NT, WA, MB, IRL). In addition, the implementation plan in IRL included the specific personnel responsible for achieving each action. In QLD, WL and TAS, strategies were prioritised but these were not associated with a specific timeframe (see Table 4).

Frameworks differed in their plans for how implementation would unfold overall and in the actors responsible for implementation (see Additional file 2 for further detail). QLD and TAS described implementation as a ‘dynamic’ process which would occur in the health service over time and would be flexible to challenges arising. The TAS framework encouraged services and workers to incorporate aspects of the framework and resources provided within the document. Actors involved in implementation were not detailed in NT though it was noted that a more detailed plan was expected to follow the framework document. A subsequent document was not identified through our search strategy. In SL, the Long Term conditions Alliance Scotland was to lead implementation including oversight of SMS provision and funding. WL identified leadership roles necessary to oversee framework implementation while MB identified primary care networks as facilitating implementation through various actions.

WA and IRL frameworks provided most detail on structures to support implementation. WA prioritised establishing a reference group and steering committee and partnership and funding strategies. In IRL a national governance structure was outlined detailing participation from each geographical area, the establishment of new roles and a group to oversee implementation, toolkits for implementation and the use of financial incentives.

#### Evaluation

All frameworks included some recommendation for evaluation. Evaluation outcomes were outlined for specific time framed actions in WA and NT. In IRL, process measures were outlined to evaluate the initial phase of implementation. Detailed plans were not outlined for other frameworks (Table 4). However, some information was provided on approaches such as: evaluation using existing reporting systems (TAS, IRL) or developing additional systems (IRL); incorporating outcomes as part of other evaluations such as evaluations of primary care (MB) or the broader chronic disease management strategy (QLD, NT); using a specific recommended framework for evaluation (SL) or an evaluation strategy being developed by each service area (TAS). SL emphasised an intent to use evaluation as a criterion for funding.

## Discussion

In this cross-country comparative document analysis, we examined SMS frameworks for chronic disease in OECD countries, guided by the Health Policy Triangle. A search of grey literature sources and government websites identified eight frameworks published from 2008 to 2017. Unsurprisingly, the rising burden of chronic disease was the primary driver for the development of each framework. The scope of frameworks varied with some targeting chronic disease prevention and health promotion through to complex disease management. The range of chronic diseases also differed across frameworks. Few frameworks considered multimorbidity, though many individuals have more than one chronic disease and this is understood to pose particular challenges for self-management [34].

Commonalities across frameworks were noted in the defining features of SMS and the goals of frameworks. A consistent pattern across the frameworks was that SMS involved empowering patients to actively manage their chronic disease in collaboration with HCPs. It is encouraging to find some commonalities on what SMS requires of HCPs and how SMS is defined given that there is little conceptual literature on SMS and in recent years it has been noted as a developing concept [35]. There were also similarities in the courses of action prioritised to enhance SMS such as the use of SMS programmes by people living with chronic disease, training of health professionals, and raising awareness about SMS services. These similarities may be somewhat attributed to countries drawing on previously published SMS frameworks and using literature to inform framework development. While only some frameworks explicitly stated the use of literature and evidence to inform frameworks, most appeared to reference literature. This suggests a commitment to underpin SMS health policy with evidence though it is noted that there are barriers to the use of evidence in policy making which require further attention [36].

There was also variation across frameworks. Differences were noted in the contents of frameworks, including the courses of action to facilitate SMS. For example, the Irish framework placed greater emphasis than some other frameworks on the need for generic and disease-specific supports and how these would integrate to provide comprehensive SMS [8]. Additionally, different strategies were used to target the appropriateness and accessibility of SMS for individuals and cultural groups across frameworks. Differences in courses of action may be to some extent attributed to inconclusive evidence on SMS approaches [5, 20]. Both the process of tailoring SMS and the combination of disease-specific and generic supports in this process have been highlighted to require further research [37]. The need for further evaluation of SMS interventions and approaches was identified in the frameworks. Thus it is possible that some of the variation in frameworks was due to the ambiguity of evidence in relation to optimal approaches to SMS. This underscores the importance of additional research on SMS approaches.

Only half of the frameworks included a high level implementation plan, that is, strategies associated with specific timeframes for which they need to be achieved. This is surprising given the emphasis placed on the need for a strategic implementation process relating to national health policy and health service reform [21, 25] as well as research indicating that SMS involves comprehensive and sustainable approaches [35]. The WHO points to the importance of being comprehensive in considering how framework changes need to be supported through health workforce resources, infrastructure, financing and governance [21]. While all frameworks attended to structural and cultural components of organisational readiness for change [24, 38] in some form, there was variation in the extent to which personnel and infrastructure for implementation were addressed, with IRL [8] and WA [26] most comprehensive in this area. Furthermore, our analysis found that the scope of chronic diseases covered by frameworks may be linked with the specificity of the implementation plan. Two frameworks (WA [26], IRL [8]) with detailed implementation plans and timelines explicitly targeted the least number of chronic diseases. The framework targeting the widest range of conditions included comparably less information on specific actions and timelines for implementation (TAS [32]). It is expected that frameworks with more specific detail and associated time framed implementation plans which are strategic and comprehensive would be successfully implemented [25].

Some frameworks allowed for greater local flexibility than others. Two frameworks (WA [26], IRL [8]) were very specific in outlining a governance structure to facilitate implementation of SMS. Others did not specify national governance structures and emphasised local flexibility, for example, services and providers choosing the resources and models appropriate to them (QLD [7], TAS [32]). Research from other policy areas has identified a need for balance between allowing local flexibility in implementation and consistent mechanisms of accountability at the broader governing level [39]. However, the optimal balance for the delivery of SMS through a national health system is unknown and has yet to be researched.

Differences in flexibility and courses of action may also be related to design of the healthcare system within a country as has been found in the case of chronic disease management approaches [16, 17]. Limited detail was provided on the healthcare systems and their influence on SMS within the framework documents. Within the policy documents, it may be useful to include information on pertinent contextual factors which influenced framework development to allow policymakers to consider context when drawing on aspects of policy from other countries. The authors are currently planning further research to examine the experience of implementing SMS frameworks. We will seek to identify lessons for implementing SMS frameworks across countries and also the context specific challenges and opportunities which affect implementation.

The involvement of actors varied across frameworks. Some documents did not detail consultation processes or actors involved. Where actors were detailed, it commonly involved health professionals and managers. Contrary to the emphasis on PPI in health strategies [22], direct involvement of people with chronic disease appears to have been uncommon. The potential benefits of involving a diverse range of actors who will implement and benefit from the framework suggest that this is a worthwhile endeavour which may enhance the success of a health policy and intervention [22]. It is noteworthy that two frameworks which documented the involvement of a large range of actors including consumers of care also have highly detailed plans for implementation (WA [26], IRL [8]). This points to some potential advantages of PPI in developing a policy implementation plan, though the exact role of actors and their contribution to the policy process requires further research.

### Strengths and limitations

Use of a policy framework and information categories to guide data collection are strengths of this study. The consistent application of a framework across documents from different health systems, as in our analysis, enhances the reliability of cross-country comparisons [40]. The Health Policy Triangle [12] facilitated a comprehensive document analysis of SMS frameworks as an initial step to understanding their context, content, actors and processes. Other frameworks for examining policy [41] and theories of the policy process [42] were considered to be more narrow in scope, focusing on either the contents of policy or the actors/processes and requiring information beyond that which is provided through policy documents. It has been recognised that there is overlap between the components of the Health Policy Triangle [13]. However, specific categories for extraction within each component of the Health Policy triangle facilitated analysis of key details in this work. Thus the framework provided a useful means of classifying information.

The grey literature search strategy, which was systematic and transparent is a strength of this study. Grey literature searching can be challenging in the absence of a ‘gold standard’ systematic method. We drew on standards and methods proposed in previous work [28], therefore conducting a rigorous and robust method of searching web-based sources of grey literature. However, there were some limitations in the grey literature search. The lack of ability to automatically export all documents meant that only one researcher, instead of two, carried out the initial phase of screening. In addition, weaknesses in archiving various details of documents as well as the means by which some search engines develop relevance ratings may have affected the retrieval of relevant documents [28].

The search was limited to documents from OECD countries and documents available in the English language. While, eight countries might be considered a limited representation of national SMS frameworks, these may largely represent health systems which have produced system-wide SMS policy documents. Language is unlikely to have severely restricted the search given the publication of some health policy documents from non-English speaking countries through English and the dearth of documents identified in English speaking health systems such as those in the United States. Countries which are not represented may have taken different policy approaches to chronic disease management with a strategy for SMS integrated into these policies rather than having a national strategy specifically for SMS in healthcare. Thus the findings of our analysis compare national SMS frameworks for chronic disease and provide a platform for further research on the operationalisation of SMS frameworks for chronic disease management.

## Conclusions

This study identified eight policy documents developed by national and state health departments in OECD countries over the past 10 years which aim to improve the provision of SMS for chronic diseases. This study served to illuminate and compare the contents and processes of existing policy for chronic disease SMS, an area which was not previously explored. Countries at early stages of SMS policy development and implementation can draw on this study to inform their national strategies for chronic disease healthcare. While there was evidence that SMS frameworks in some countries drew on the work of other countries, there was little evidence of active engagement between policy makers in different countries to learn from each other. Given that health policies are not always implemented as planned; it is important to understand the success factors or barriers relating to implementation so that proposed plans for SMS can be operationalised in ways that contribute to reducing the burden of chronic disease. Factors were identified in this study which could influence the implementation of SMS policies. Further research needs to examine the influence of these factors.

## Additional files

Additional file 1: Electronic Search Strategy. This file depicts the search strategy used for different grey literature databases and search engines. (DOCX 16 kb)

Additional file 2: Data Extraction Table. This file includes the data which was extracted from documents in this study and forms the basis of the results section. (DOCX 35 kb)

## Abbreviations

HCP: Healthcare Professional
IRL: Ireland
IT: Information Technology
MB: Manitoba
NT: Northern Territory
OECD: Organisation for Economic Co-operation and Development
PPI: Patient and Public Involvement
QLD: Queensland
SL: Scotland
SMS: Self-management Support
TAS: Tasmania
WA: Western Australia
WHO: World Health Organisation
WL: Wales
