# Supplementary material for: Frameworks for self-management support for chronic disease: a cross-country comparative document analysis
Source: BMC Health Serv Res. 2018 Jul 25;18:583. doi: 10.1186/s12913-018-3387-0 (PMC6060470; doi:10.1186/s12913-018-3387-0)
Supplement: Supplementary file 1 — Electronic Search Strategy. This file depicts the search strategy used for different grey literature databases and search engines. (DOCX 16 kb) [file 12913_2018_3387_MOESM1_ESM.docx]

**Additional File 1: Electronic Search Strategy**

**Part 1: Grey Literature**

The search strategies used for grey literature sites are outlined in the table below. All retrieved documents were screened.

| **Name Grey Literature Database** | **Search Terms** | **Searched In** | **Limits applied** | **No. documents Retrieved** | **No. potentially relevant** |
| --- | --- | --- | --- | --- | --- |
| BIREME | self-manag* | Title, abstract, subject | English language, 2007-2017,  Exclusion of articles | 6 | 0 |
| OECD iLibrary | self-manag* | Title, abstract | English language, 2007-2017 | 13 | 0 |
| Open Grey | self-manag* | NA | English language | 164 | 0 |
| Grey Literature Report | self-manag* | NA | NA | 23 | 3 |
| Canadian Electronic Library | self-manag* | NA | English language, 2007-2017 | 276 | 4 |
| Analysis and Policy Observatory | self-manag* | NA | NA | 39 | 0 |
| WHO IRIS | Self-manag- | NA | NA | 6 | 0 |
| OAIster | “self-manag*” AND (policy OR policies OR framework OR guideline OR model OR strategy OR strategies OR standard) AND ("long-term condition" OR "chronic condition" OR "chronic illness" OR "chronic disease" OR "non-communicable disease") | Keyword | English language, 2007-2017 | 203 | 0 |

**Part 2: Advanced Google Search**

The search strategy for the Advanced Google search is detailed in the table below. The first 10 pages of each search were screened (overall 500 results screened).

| **#** |  | | **Terms Searched** |  | | **Total search yield** | **New potentially relevant records** |
| --- | --- | --- | --- | --- | --- | --- | --- |
| 1 | self-manage OR self-management OR self-managing | policy OR policies OR framework OR guideline OR model OR strategy OR strategies OR standard | | | “long-term condition” | 31,600 | 8 |
| 2 | self-manage OR self-management OR self-managing | policy OR policies OR framework OR guideline OR model OR strategy OR strategies OR standard | | | “chronic condition” | 75,800 | 14 |
| 3 | self-manage OR self-management OR self-managing | policy OR policies OR framework OR guideline OR model OR strategy OR strategies OR standard | | | “chronic illness” | 290,000 | 2 |
| 4 | self-manage OR self-management OR self-managing | policy OR policies OR framework OR guideline OR model OR strategy OR strategies OR standard | | | “chronic disease” | 676,000 | 1 |
| 5 | self-manage OR self-management OR self-managing | policy OR policies OR framework OR guideline OR model OR strategy OR strategies OR standard | | | “non-communicable disease” | 11,200 | 0 |

**Part 3: Ministry and Department of Health websites for OECD countries**

Searched using the terms ‘self-manage’ and ‘self-managing’ and ‘self-management’ within the website search bar and hand searched where there was no search bar.
