# Supplementary material for: Frameworks for self-management support for chronic disease: a cross-country comparative document analysis
Source: BMC Health Serv Res. 2018 Jul 25;18:583. doi: 10.1186/s12913-018-3387-0 (PMC6060470; doi:10.1186/s12913-018-3387-0)
Supplement: Supplementary file 2 — Data Extraction Table. This file includes the data which was extracted from documents in this study and forms the basis of the results section. (DOCX 35 kb) [file 12913_2018_3387_MOESM2_ESM.docx]

**Additional File 2: Data Extraction Table**

| **Title,**  **Year,**  **Country/State,**  **Authoring Bodies** | **Context** | **Content**   1. **Scope of chronic disease (CD) conditions supported** 2. **SMS defining features** 3. **Goal of framework** 4. **SMS priorities/courses of action** | **Actors**  **(Framework Development only)** | **Process**   1. **Development** 2. **Proposed Implementation** 3. **Proposed Evaluation** |
| --- | --- | --- | --- | --- |
| Queensland Strategy for Chronic Disease 2005-2015: Framework for self-management 2008-2015,  2008,  Queensland,  Queensland Health | Burden of chronic disease on individuals, community and health service due to ageing population, increased lifestyle and risk factors, improved survival from advances in treatment, increased prevalence of disease. CD accounting for one third of preventable deaths. Priority of state chronic disease strategy and broader Australian health policy.  CDSMS provision before framework not reported. | 1. Type 2 diabetes mellitus, chronic respiratory disease (COPD and asthma), renal disease, CVD (coronary heart disease, heart failure and stroke), depression and anxiety. Also targets lifestyle and behavioural risk factors. 2. Process involving multilevel changes in healthcare systems and the community. 3. ‘To empower and prepare individuals to manage their health and health care’ p.5 4. 21 strategies overall. Priorities for future investment:  - SMS suitable for minority groups, different cultures, languages, remote people - education and training for service providers and minimum standards of practice - Self-management (SM) and CD education for individuals, carers and significant others and evidence-based programs - capacity building in communities - social marketing campaign to raise awareness - consumer personal health record | CD Strategy Implementation Team developed framework in consultation with the Queensland Self-Management Alliance. Consultation with government departments, NGOs, professional bodies, the private sector, consumer advocacy groups, HCPs universities and national and international experts. | 1. Consultation with actors, best available evidence and existing frameworks. Consultation involved forums, an electronic questionnaire, feedback on drafts such as progress reports and meetings with key stakeholders. 2. Implementation plan not reported. Implementation recognised as a dynamic process aided by local, regional and national initiatives (new and existing) across sectors and delivery contexts. Implementation proposed to be flexible to challenges arising within health environment. 3. Evaluation identified as a priority. Evaluation part of broader Queensland CD strategy. Evaluation strategy developed by University body ‘in consultation with key stakeholders’ p.14 though this strategy is not detailed. Recommends that initiatives should be evaluated from perspective of consumers. |
| Gaun Yersel: The Self Management Strategy for Long Term Conditions in Scotland,  2008,  Scotland,  Long-term conditions alliance Scotland (LTCAS) and Scottish Government | Growing challenge of CD, economic and social cost, impact on a person’s wellbeing. SM a component of Scottish health policy.  CDSMS before not adequate: influenced by internal and external pressure, failing to place person at centre of care. | 1. Not explicit. Reference made to conditions such as diabetes, dementia, mental health conditions, epilepsy, arthritis and breast cancer and multimorbidity. Emphasis on care for stages of disease from diagnosis onward. 2. The collaboration of the person and appropriate individual. Provided by HCPs, social care providers and carers. 3. ‘To work towards a situation in which people living with long term conditions have access to the support they need to successfully manage their condition’ p.5 4. 38 aims to achieve effective self-management at the level of the individual, unpaid carers, voluntary sector, health service, local authorities, government, employers. Strategies are not prioritised. Common courses of action: personalised action plan/appropriate SMS, awareness raising campaigns, coordination of services, building evidence base, ensuring funding packages and incentives, planning long term. | Working group made up of 6 representatives from different health and CD organisations. Input from LTCAS. Emphasised that the framework is developed from the perspective of patients. Further actors not reported. | 1. Strategy developed by working group with input from LTCAS who facilitated consultation on the draft strategy. Proposed to build on work of voluntary organisations over past years. 2. Implementation plan not reported. LTCAS taking lead. LTCAS establish advisory board and personnel to map SMS provision, feedback information and oversee funding. Recommended to establish specific fund to support development of SMS with widely consulted upon criteria. Organisations to make bids for SMS initiatives. 3. Evaluation plan not reported. An evaluation framework is recommended for use. Ongoing evaluation proposed as criteria for securing funding for SMS initiatives. |
| Improving Health and Wellbeing in Wales: A Framework for Supported Self Care,  2009,  Wales,  Welsh Assembly Government | Growing prevalence of CD, economic cost, demand on health and social care services. Impact on wellbeing, barriers to return to work, a policy priority.  Before CDSMS services not aligned with SM concept. Lack of coordination across organisations and locations. Public and professionals often not aware of services. Provision of written and verbal information lacking and attendance at SM training very low. Evidence of clinical application of SMS but no whole systems approach. | 1. Not explicit. Reference to a range of conditions such as diabetes, CVD, cancer, depression. Spans continuum from health promotion and disease prevention to complex cases of chronic conditions including multiple conditions. 2. Supported self-care: shared responsibility between individuals and HCPs. Access to the relevant resources e.g. education, and support networks. 3. Not explicit. Coordinated services to support self-care. 4. 16 recommended actions; 8 national and 8 local. Four ‘key elements of self care support’:  - Information about services, how to access it, and how to self care/ self manage; available in various formats. - Skills Training for the public, patient (general skills, disease-specific skills and education) and professionals. - Peer Support Networks (increasing awareness of availability and signposting) - Assistive Technologies e.g. reminders, self and tele-monitoring, telephone follow-up | Not reported. | 1. Not reported. Visible that document informed by research evidence and local surveys of SMS service provision. 2. Implementation plan and timelines not reported. Some process recommendations e.g. identifying a national and local lead, ensuring an infrastructure for local delivery of training programs and using incentives and continuing professional development to ensure HCPs adopt training and standards; improving access to information and signposting by NHS Wales. Consideration given to leadership and collaboration across stakeholders and organisations. 3. Recommended to evaluate four elements of framework – self-care interventions – and evaluate at local level. Evaluation plan not reported. |
| WA Chronic Conditions Self-Management Strategic Framework 2011–2015,  2011,  Western Australia,  Department of Health Western Australia | Health system reform, burden of CD especially societal burden, with affected individuals less likely to participate in the workforce. Priority of state chronic disease and broader health policy.  CDSMS provision before framework not reported. | 1. CVD, type 2 diabetes mellitus, chronic renal disease, chronic musculoskeletal conditions and chronic respiratory disease. Noted that applicable to other conditions. Applies to ‘continuum of care’. 2. Strategies carried out by HCPs, carers, organisations and systems to assist individuals in SM. 3. ‘The active participation by people in their own health care within their communities’ p.5. 4. 5 ‘essential elements’ aligned to objectives, with strategies:  - Culture: promoting supportive attitudes through endorsing strategic framework, partnerships and networking - Awareness: promoting SM through marketing strategy, website, easy-to-navigate referral pathways - Services: access to quality coordinated SM programmes and services through flexible delivery, community networks - Knowledge and Skills: building capacity through competency framework for HCPs, training, professional development, curriculum, mentoring - Tools and Resources: accessible information through website for all stakeholders and quality improvement framework | Strategy Review Group included CD consumers, carers, managers, policy developers, service providers, NGOs, researchers, CD SM educators. | 1. Consultation included an electronic qualitative survey, workshops with Strategy Review Group and feedback on draft. 2. Actions associated with short term (2 years) and longer term goals (2-4 years). Initial implementation plans focused on providing infrastructure for implementation. Developing a plan for the strategic framework is a short term goal. Scoping document provides structure for implementation. Process involves establishing a reference group and steering committee and partnership and funding strategies. Implementation to be tailored to local needs and resources. 3. Evaluation Plan reported including outcomes specified according to short and long term goals and addressing the 5 ‘essential elements’ of the framework. Reference Group and Steering committee responsible for identifying evaluation priorities and KPIs. Framework to be reviewed in line with new evidence. |
| Self-Management and Primary Care in Manitoba: The Way Forward,  2012,  Manitoba,  Manitoba Health Canada. | Growing prevalence of CD, demands on health system. Large spending on individuals with CD and indirect costs such as loss of economic output. Impact on individual and community wellbeing.  Before CDSMS limited by inadequate provision by primary care services, especially in remote areas, lack of provider understanding of programs and self-assessment of training needs. Difficulty in attracting certain groups. Acute care setting overburdened. Low levels of SMS such as being asked about goals, receiving written information, referral to group or program. | 1. Not explicit. A range of chronic conditions referred to and some highlighted: coronary heart disease, stroke, diabetes, asthma and COPD, osteo- and rheumatoid arthritis. Spans continuum of care, from identification of risk to CD management. 2. Resources, programs, services, or tools to support individuals in SM. 3. Not explicit. Strengthen SM and SMS. 4. 6 recommended actions for first year and 3 for second year. Actions at system/provider/individual level:  - Narrowing knowledge on appropriateness of SMS initiatives for different groups - Integration and promotion of SMS within primary care - Increase capacity and reach of teleCARE SMS - Existing services sustained and enhanced - Online health portal to provide tailored SMS - Strategies to increase provider awareness of SMS - Focus on high risk groups | Not reported. | 1. Review of evidence of effectiveness from other areas, as well as reviewing current provision and effectiveness of SMS in Manitoba and Canada. 2. Implementation plan not reported. To be facilitated by Primary Care Networks through increasing IT connectivity, group sessions and outreach. Specific recommendation to increase provider awareness to be achieved through existing partnerships with universities, regional health authorities and NGOs. 3. Evaluation plan not reported. New supports to be evaluated as part of a Primary Care Network evaluation. Comprehensive evaluation framework for SMS initiatives recommended in order to learn about efficacy and group specific uptake. |
| A Framework to support Self Management,  2012,  Tasmania Australia,  Department of Health and Human Services Tasmania | CD contributes largely to disease burden. Tasmania has highest rate of chronic conditions of the Australian states. Framework forms part of wider Chronic Disease Strategies.  CDSMS provision before framework not reported. | 1. Diabetes, CVD, asthma, COPD, cancer, communicable diseases including hepatitis B and C and HIV/AIDS; neurological conditions, chronic disability from injury, chronic pain, arthritis, inflammatory bowel disease, lifestyle risk factors, mental health conditions. Spans continuum of care moving from acute to a continuous approach in relation to prevention and health promotion. 2. Support to individual and family/carer by HCPs and the system. 3. ‘To support health and human services to implement self management approaches to achieve better outcomes for people living with chronic conditions’ p.4. 4. 26 strategies under 4 objectives. SMS priority areas:  - Education and training for HCPs including communication skills, SM programs available, training options available, evidence-based clinical practice, range of SM strategies - Development of systems including integrating SMS into practice, minimum standards of practice, multidisciplinary working, SM programs and community support, providing a range of flexible resources - Connecting people and resources through communication processes. - Benchmarking and monitoring to build a local evidence base. | SM Framework Reference Group and Chronic Conditions Clinical Network aided framework development. Members of the Reference Group listed and appear to involve HPs and programme managers. Exact contributions of organisations and individuals not detailed. | 1. Detail on process of actor involvement not reported. Framework supported by a background paper and builds on frameworks from other geographical areas. Priority areas were identified during consultation. 2. Implementation plan not reported. It will be a ‘dynamic process’ which occurs in different ways over time across the state. Services and workers encouraged to incorporate elements of framework in practice, using resources provided while other aspects will be implemented organisations as part of other framework initiatives. 3. Evaluation plan not reported. Research and evaluation are prioritised as initial implementation areas. Evaluation should form part of other reporting structures, and be conducted regularly. Approach to be developed by individual service area. |
| The Northern Territory Chronic Conditions Self-Management Framework 2012 – 2020,  2012,  Northern Territory,  Northern Territory Department of Health | CD large contribution to disease burden, mortality, use of hospital resources. Framework part of broader CD strategy.  CDSMS before was uncoordinated and provided by different government and community organisations and NGOs. Challenge in adopting SMS in practice, particularly for Aboriginal clients in remote areas. | 1. Applies to all. Focus on CVD, rheumatic heart disease, type 2 diabetes, chronic airways disease, chronic kidney disease, chronic mental illness, cancers associated with common risk factors for other chronic conditions. Considers risk factors for diseases. 2. Actions of HCPs, carers and health system to assist client to SM. 3. ‘Health services that promote and support self management and empower individuals with chronic conditions to actively participate in their own health care’ p.14 4. 10 strategies and associated actions for implementation under 3 objectives. Strategies and actions:  - Build culture: framework endorsed by stakeholders, modify guidelines/standards, SM network, clear referral pathways - Access to quality services: SM recorded in electronic client record, develop HCP skills in evaluating SM initiatives, support HCP access to evidence based practice, accessible information through various mediums - Build capacity: managers facilitate training of HCPs and tools to support culturally sensitive SMS, promote SMS and groups | Working Group made up of HCPs providing SMS. Consultation: representation of government, non-government and Aboriginal community controlled health service providers. HCPs (14) and consumer groups (5) involved in Focus Group. | 1. Working Group developed framework. Focus groups, in urban and remote communities. Draft framework sent for comment to all consulted. Used evidence of experience and frameworks in other geographical areas. 2. Includes implementation plan. 10 strategies identified for implementation which are linked with a number of actions to be taken in short (2012-2014), medium (2015-2017) and long term (2018-2020). Those responsible for actions not detailed. More detailed plan expected to follow. 3. Performance measures specified for each action and associated timeframe. Framework to be evaluated with broader CD strategy, will report on identified performance indicators. |
| Living Well with Chronic Conditions: Framework for self-management support,  2017,  Ireland,  Health Service Executive | CD contributes ill health, mortality, health service activity and expenditure. Prevalence forecast to increase. SMS a priority of WHO and national policies.  CDSMS services before not meeting need, variable across locations. Low rates of SMS such as written information on how to manage condition, written care plans, and HPs asking about goals when making plan; personalised care planning not facilitated in primary care. | 1. COPD, Asthma, Diabetes and CVD (ischaemic heart disease, heart failure, stroke, hypertension), multi-morbidity. Focus largely on SM of mentioned conditions. 2. Education and interventions (psychological and social support, support for SM of physical care, self-monitoring, and adherence strategies) to increase patient skills and confidence to SM, systematically provided. 3. Not explicit. Enhance provision of SMS. 4. 37 actions for implementation under 6 areas.  - Government and policy: appoint roles/structures to oversee implementation - Partnerships: between HCP and patient; health service and voluntary/community organisations - Training and Staff Management: education in undergraduate curriculum and part of professional development - Operational Planning and Resources: map and increase provision of evidence based SMS programmes; toolkits for implementation; resources factoring in health literacy - Cross divisional and wider systems: SMS communication plan, IT systems to support implementation - Monitoring and evaluation | Working group: health division and programme managers/coordinators and researcher. Advisory group: 36 individuals including HCPs, representatives of patient and chronic disease-specific organisations, two persons with CD. Consultation on draft: all the above and representatives from health service senior management, college of GPs and Department of Health. | 1. Working group developed framework supported by advisory group. Consultation on draft framework through focus groups and interviews. Wide range evidence used: Health Technology Assessment, literature, international policy documents, survey of existing service provision, findings based on national consultation with patients. 2. Implementation plan included with timelines. Priorities for initial implementation and plans for Phase 2. Governance structure outlined: national programme lead and National Oversight Group (with patient representation), named leads at local level as well as SMS coordinators (mapping SMS provision and creation of directories), partnerships between organisations and financial incentives to be used. 3. Some aspects of future evaluation detailed: harness existing systems of data collection and develop further if necessary, identify KPIs (process and outcome), evaluate over long term and from multiple perspectives. Process measures outlined for initial phase. |

**Abbreviations**

CD: chronic disease; CDSMS: chronic disease self-management support; COPD: chronic obstructive pulmonary disease; CVD: cardiovascular disease; HCP: healthcare professional; IT: information technology; KPI: key performance indicator; LTCAS: Long-term Conditions Alliance Scotland; NGO: non-governmental organisation; SM: self-management; SMS: self-management support; WHO: World Health Organisation.
